# ESI-MS Analysis of Thiol-yne Click Reaction in Petroleum Medium

**DOI:** 10.3390/molecules26102896

**Published:** 2021-05-13

**Authors:** Evgeniya S. Degtyareva, Julia V. Burykina, Valentine P. Ananikov

**Affiliations:** Zelinsky Institute of Organic Chemistry, Russian Academy of Sciences, Leninsky Prospect 47, 119991 Moscow, Russia; ed@ioc.ac.ru (E.S.D.); ivanova@ioc.ac.ru (J.V.B.)

**Keywords:** click reaction, thiols, ESI-MS, hydrothiolation, catalysis, crude oil, derivatization

## Abstract

Petroleum contains a large number of heteroatomic compounds, but today, most of them are not efficiently utilized. The constant development of the sustainability concept recalls for rethinking the usage of fossil resources with improved chemical utility. In order to initiate research aimed at involving active petroleum compounds in chemical transformations, a new analytical method for product detection is needed. Here, we study the click reaction of thiols with alkynes, leading to the formation of α-vinyl sulfides directly in the petroleum environment. The reaction was carried out using an (IMes)Pd(acac)Cl catalyst, which demonstrated tolerance to petroleum components. In this study, the concentration of thiols ranged from 1 M to 0.01 M (from 8% to 0.1%). To detect products at low concentrations, a special alkyne labeled with an imidazole moiety was used. This approach made it possible to observe the formation of vinyl sulfides by electrospray ionization mass spectrometry (ESI-MS), which provides an opportunity for further optimization of the reaction conditions and future developments for the direct involvement of oil components in chemical reactions.

## 1. Introduction

Crude oil is one of the most complex mixtures in the world, with a dynamic range of 10,000–100,000 compounds with different structures and properties. At the same time, the chemical complexity of crude oil necessitates to reconsider its role from fuel to a multipurpose chemical feedstock. Particular attention should be paid to heteroatom-containing organic compounds (N, O, S), which are present in crude oil in up to 10% [1]. The most common heteroatom in oils is sulfur, which is found in the form of dissolved elemental sulfur, hydrogen sulfide, thiols, sulfides, disulfides, and thiophene derivatives, as well as complex compounds containing various combinations of S, O, and N atoms [2,3]. The average amount of sulfur in oils is approximately 3–4%. Thus, the efficient use of oil as a global natural source of S-functionalized substrates in chemistry may become a new round of development in the field of petrochemistry.

The addition reaction is an elegant, atom-efficient way of producing a variety of sulfides, which are in great demand in material, polymer, and chemical science [4,5,6,7,8,9]. Investigation of the procedure for the direct involvement of the components of crude oil in chemical syntheses requires an efficient methodology of component assignment. To date, a number of analytical methods have been developed for the detailed analysis of oil components, where high-resolution mass spectrometry (HRMS) was found to be an excellent approach for rapid analyzes and classification of tens of thousands of components [10]. In fact, mass spectrometry is the main method for the identification and determination of individual structural entities in crude oil [11,12,13,14].

Electrospray ionization mass spectrometry (ESI-MS) is a “soft” ionization technique due to very little fragmentation. After discovery by Fenn and coworkers, the method was applied to reveal the composition and nature of complex organic mixtures [15]. The principle of the ionization process is the formation of microdroplets including charged particles. The pioneering work of Zhan and Fenn demonstrated the use of ESI-MS for the analysis of crude oil, jet fuel, gasoline, and coal [16]. The application of the ESI source with ultrahigh resolution mass analyzers provides an excellent opportunity to resolve multiple ions and to simultaneously identify each component, thereby expanding the horizons of in-depth knowledge of the chemical composition of oil at the molecular level [17,18,19].

Indeed, the topic of oil characterization is of great interest. Since the characterization of oil is a very complex task, each developed technique demonstrates selectivity in relation to a certain class of compounds [20,21,22]. In this article, we begin the search for a new approach towards the estimation of sulfur-containing species that are able to participate in a functionalization reaction. The target substrates are thiols and the products of their interaction with alkynes—vinyl sulfides. This process usually occurs via radical, nucleophilic, or catalytic pathways [23,24,25,26]. Today, systems based on Cu-, Ni-, or Pd-complexes find wide application in the hydrothiolation process [27,28,29,30] as well as other reactions of C-S bond formation [31,32,33,34]. Taking into account the high practical demand in metal-catalyzed methodology, a catalytic reaction driven by transition metals was studied. In the current work, the choice of the reaction is dictated by the ultrahigh selectivity of the system with respect to S-containing species.

In the present study, we demonstrate progress in two challenging directions. First, we show that a catalytic process involving oil components is directly possible, thus highlighting the principal possibility of simultaneous using of oil as a reagent and solvent. Second, we report a practical approach of using ionizable acetylenic tags to enhance the analytic methodology to monitor click reactions in oil media.

## 2. Results and Discussion

The study of reactions in crude oil places particular demands on catalysts and substrates. This article will discuss the possibility of direct utilization of thiols in petroleum media for the synthesis of vinyl sulfides by metal-catalyzed thiol-yne click reaction. Naturally, thiols are concentrated in light petroleum fractions with the boiling point up to 300 °C in the form of linear, branched, or cyclic aliphatic species and thiophenol [2,3]. Therefore, the catalyst of choice must be active towards aliphatic thiols and, of course, must be tolerant of other compounds in the oil.

**Figure 1:**
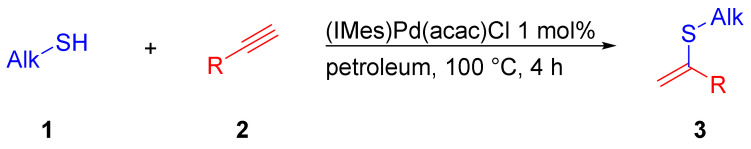
Model click reaction between aliphatic thiol (**1**) and alkyne (**2**) catalyzed by the Pd/NHC complex and resulting in the formation of vinyl sulfide (**3**).

The model synthesis of aliphatic thiol addition (**1**) to alkyne (**2**) was carried out directly in petroleum; therefore, the catalyst should preserve activity in a nonpolar medium. Additionally, the process should be tolerant to the presence of water traces with no necessity for an inert atmosphere. Summarizing the above parameters, we used a palladium complex with an N-heterocyclic carbene ligand (Pd-NHC) for the present study (Figure 1) [35].

The low concentration of thiols in the solution and the complex composition are the first stumbling blocks to use natural petroleum for chemical synthesis. In order to tune the reaction conditions to maximize the product yield, a method of fast and convenient analysis of the reaction mixtures needed to be developed.

As discussed above in the Introduction, the most convenient analytical method for solving this problem is ESI-HRMS. The amount and type of detected compounds are affected by the sensitivity of the instrument, as well as various discrimination and ion suppression effects during the ionization process [36]. For the studied process, this method provides (1) the ability to detect tiny amounts of products due to its high sensitivity and (2) uninformative signals from hydrocarbons (predominant chemical class in oils) due to their poor ionization ability; thus, the resulting mass spectrum is easy to interpret.

**Figure 2:**
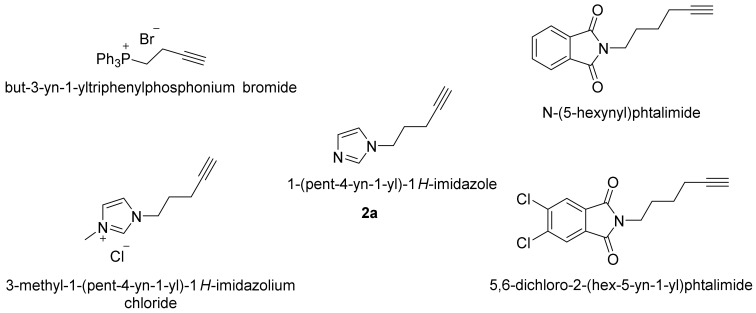
Selected alkynes tuned for ESI-MS detection used in this study.

To enhance the level of ionization of the reaction products, specific alkynes with mass spectrometric targets and well ionizable groups were used (Figure 2). Two alkynes were ionic compounds (triphenylphosphonium and imidazolium), and the others contained ionizable imidazole or phthalimide moieties. (5,6-Dichloro-2-hex-5-yn-1-yl)phthalimide contained two chlorine atoms to enhance ion recognition by the characteristic isotope distribution in ESI-HRMS. The indicated alkynes were synthesized and tested in a model reaction with a 0.1 M solution of 1-pentanethiol (**1a**) in petroleum ether.

The best results were obtained in the reaction with 1-(pentyn-4-yn-1-yl)-1H-imidazole (**2a**): the presence of imidazole did not adversely affect the activity of the catalyst (Table S1, Supplementary Materials) but allowed us to obtain high intensity signals in the mass spectrum (Figure S1, Supplementary Materials). Highly polar 3-methyl-1-(pent-4-yn-1-yl)-1*H*-imidazolium chloride and but-3-yn-1-yltriphenylphosphonium bromide were found to be insoluble in petroleum ether, which resulted in the formation of product 3 in trace amounts (Table S1, Supplementary Materials). Despite the highest phthalimide-based alkyne conversion into product 3, analysis of the results of ESI-MS studies unexpectedly showed low ionization activity (Figure S1, Supplementary Materials). Summarizing the obtained results, imidazole-containing alkyne **2a** was chosen for further study.

It is extremely challenging to carry out selective reactions in petroleum media because many components can passivate or poison the catalyst. To ensure that the catalytic cycle will not be stopped, the model reaction between thiol **1a** and alkyne **2a** was repeated directly in a petroleum medium. The utilized petroleum sample is a blended crude oil before any treatment on the oil refinery plant with density at 20 °C = 0.8518 g/mL, sulfur content = 1.342%. The thiol concentration in the model system was taken to be equal to 1 M (8%). The formation of vinyl sulfide **3a** (1-(3-(pentylthio)but-3-en-1-yl)-1*H*-imidazole) was registered by ^1^H NMR and ESI-HRMS analyses (Figures S2 and S3A, Supplementary Materials). In the next step of the study, the preservation of the catalytic system efficiency for diluted systems was verified. For this purpose, a model reaction of **1a** and **2a** in petroleum was carried out, while the concentration of substrates (thiol:alkyne = 1:1) was successively reduced from 1 M to 0.1 M, 0.05 M, and 0.01 M (Table 1, Figure S3, Supplementary Materials). The concentration of thiol 0.01 M (0.1%) simulates natural high-sulfur oils with 10% thiols with a total sulfur content of 2%. Within this concentration range, the signals in the ESI-MS positive ion mode were sufficient for reliable detection of reaction product **3a**. It is important to point out that the observed signals of **3a** corresponded to [M + H]^+^ (*m/z* 239.1576) and less typical [M − H]^+^ (*m/z* 237.1420) ions (Figure S3, Supplementary Materials). Formation of [M − H]^+^ ions was earlier observed by our group for vinylsulfides and monoselenide-substituted-1,3-dienes [25,37]. Here and further, the most intensive signals (more often [M − H]^+^) are described.

**Figure 3:**
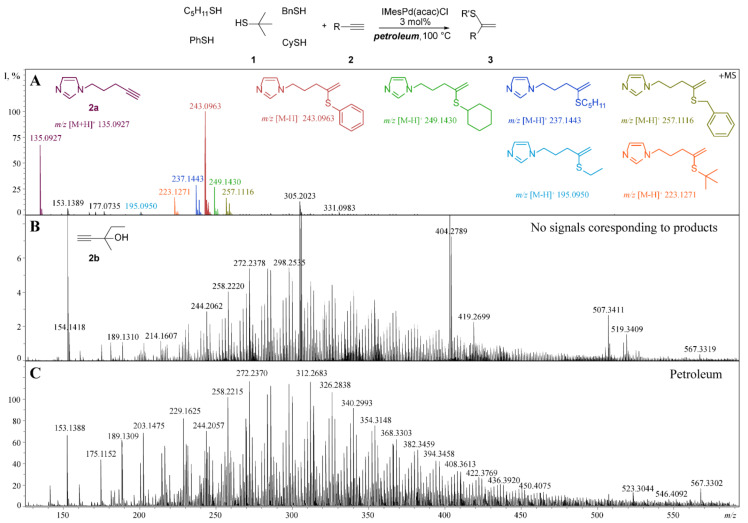
Comparison of the ionization level of reaction products **3** in the hydrothiolation process, which involved “electrospray tuned” alkyne **2a** (**A**) and in reaction with alkyne **2b** without ESI tag (**B**) carried out in the petroleum medium. The spectrum of pure petroleum is demonstrated for comparison (**C**). The ESI-(+MS) spectra were recorded in positive ion mode in acetonitrile solution.

In the next step, the scope of thiols was evaluated to estimate the acceptable range of thiol concentrations. For this purpose, a hydrothiolation reaction of imidazole-based alkyne **2a** by a mixture of thiophenol, benzylthiol, cyclohexylthiol, pentanethiol-1, and *tert*-butyl thiol was carried out in petroleum medium. The concentration of alkyne and thiols was 0.1 M. The mixture contained 1 mol% of (IMes)Pd(acac)Cl and was heated at 100 °C overnight. Afterward, the aliquot was analyzed by ESI-HRMS (×200 dilution, Figure 3, Figures S4–S9, Supplementary Materials). In the obtained spectrum, it is easy to identify the formed vinyl sulfides, since the intensity of the signals of the reaction products (≈10^6^ rel. units) significantly exceeds the intensity of oil signals (4000 rel. units). The variation in intensity level for product 3 signals in the ESI-MS spectrum is caused by different thiols reactivity. According to the earlier study, aromatic thiols typically show higher activity over aliphatic thiols in the hydrotiolation reaction catalyzed by (IMes)Pd(acac)Cl [35]; therefore, the observed intensity of 1-(4-(phenylthio)pent-4-en-1-yl)-1*H*-imidazole was the highest.

In order to demonstrate the enhancement of the ionization level in the reaction with imidazole-labeled alkyne **2a**, the reaction with 3-methyl-1-pentyn-3-ol (**2b**) was carried out. The choice of alkyne **2b** was suggested by a previous study of the (IMes)Pd(acac)Cl-catalyzed hydrothiolation reaction, where the corresponding vinyl sulfide was obtained in a high yield [35]. The analysis of the registered mass spectra for the reaction with **2a** (Figure 3A) and **2b** (Figure 3B) clearly demonstrates the advantage of the utilization of labeled alkyne: in the reaction with **2b**, a search of the product signals failed due to low intensity and the numerous overlapping petroleum components.

The initial oil sample contained methyl and ethyl mercaptanes (MeSH content = 0.69 ppm, EtSH content = 0.49 ppm); therefore, a precise analysis of the HRMS spectrum obtained from the reaction with imidazole-labeled alkyne **2a** and its comparison to the spectrum of petroleum (Figure 3A,C) was carried out. Amazingly, the signal with *m/z* 195.0950 corresponding to the product of C_2_H_5_SH addition to **2a** was found (Figure 3A, Figure S10, Supplementary Materials). Inspired by the observed product, the same experiment, though without thiol addition, was carried out. In the registered mass-spectrum (Figure S11, Supplementary Materials) ions corresponding to products of ethanethiol addition to **2a** (*m/z* [M − H]^+^ 195.0950 and *m/z* [M + H]^+^ 197.1107) and propanethiol addition to **2a** (*m/z* [M − H]^+^ 209.1107 and *m/z* [M + H]^+^ 211.1263) were found, thus demonstrating the utility of the developed procedure for naturally occurring thiols in petroleum.

## 3. Conclusions

In the current article, the opportunity to utilize minor petroleum components in organic synthesis is investigated by example of a thiol-yne click reaction. Summarizing the results of the study, we would like to highlight the following main points. First, petroleum can simultaneously be utilized as a source of active heteroatom-substituted organic compounds and as a solvent. Second, even for such a multicomponent mixture as petroleum, it is possible to find a catalyst that will be active and selective towards the desired substances and tolerant to numerous other heteroatom-containing components in the mixture. Moreover, the widely circulating opinion that multicomponent natural systems are unsuitable for synthetic applications was corrected by the demonstration of the formation of products of the thiol-yne click reaction in natural petroleum. A specially designed analytical approach based on the use of ESI-HRMS in combination with imidazole-labeled alkyne with high ionization ability was employed for improved observation of the desired transformation.

## 4. Materials and Methods

### 4.1. General Procedures

Reactions were performed in screw-capped glass test tubes with magnetic stir bars in an air atmosphere unless otherwise stated. Thiols were purchased from commercial sources and checked by ^1^H and ^13^C NMR spectroscopy prior to use. (IMes)Pd(acac)Cl, but-3-yn-1-yltriphenylphosphonium bromide, and N-(5-hexynyl)phthalimide were synthesized according to described procedures [38,39,40]. Mixed petroleum sample was used with density at 20 °C = 0.8518 g/mL, chloride content = 16.57 mg/mL, sulfur content = 1.342%, MeSH content = 0.69 ppm, EtSH content = 0.49 ppm.

NMR measurements were performed using a Bruker AVANCE 600, Bruker DRX-500, Bruker Fourier 300 spectrometers operating at 600.1 and 500.1, 300.1 and 150.0, and 125.8 and 75.5 MHz for ^1^H and ^13^C, respectively. The ^1^H and ^13^C chemical shifts were referenced to internal standards provided by the solvent or relative to TMS as an external standard for spectra in CDCl_3_.

High-resolution mass spectra were obtained on a Bruker maXis Q-TOF instrument (Bruker Daltonik GmbH, Bremen, Germany) equipped with an electrospray ionization (ESI) ion source. The experiments were performed in positive (+) MS ion mode (HV Capillary: 4500 V; HV End Plate Offset: −500 V) with a scan range of *m/z* 50–1500. External calibration of the mass spectrometer was performed using a low-concentration tuning mix solution (Agilent Technologies, Santa Clara, CA, USA). Direct syringe injection was applied for the analyzed solutions in MeCN (flow rate: 3 µL/min^−1^) for analytical characterization. Nitrogen was applied as nebulizer gas (1 bar) and dry gas (4.0 L/min, 200 °C). The spectra were processed using Bruker Data Analysis 4.0 software.

### 4.2. Synthesis of 1-(pentyn-4-yn-1-yl)-1H-imidazole (**2a**)

Imidazole (1.2 mmol, 0.0817 g), 5-chloropent-1-yne (1 mmol, 0.106 mL), and 0.12 mL of 50% KOH solution in water were mixed in a screw-cap test tube and then dissolved in 0.175 mL of DMSO. The test tube was purged with argon and closed. The reaction was carried out for 2 h at 60 °C. Then, 2 mL of water was added to the test tube, and the reaction product was extracted with Et_2_O (5 × 2 mL). The organic phase was dried over MgSO_4_, filtered, and evaporated. The pure product as a yellowish oil was obtained after flash vacuum chromatography with a 61% yield.

^1^H NMR (600 MHz, CDCl_3_) δ, ppm: 7.50 (s, 1H), 7.07 (s, 1H), 6.95–6.92 (m, 1H), 4.11 (t, *J* = 6.8 Hz, 2H), 2.18 (td, J = 6.7, 2.7 Hz, 2H), 2.06 (t, *J* = 2.7 Hz, 1H), 1.97 (quint, J = 6.7 Hz, 2H). ^13^C{^1^H} NMR (151 MHz, CDCl_3_) δ, ppm: 137.33, 129.63, 118.91, 82.15, 70.17, 45.20, 29.52, 15.44.

*3-methyl-1-(pent-4-yn-1-yl)-1H-imidazolium chloride.* 1-Methyl-1H-imidazole (2 mmol, 0.159 mL) and 5-chloropent-1-yne (2 mmol, 0.212 mL) were mixed in a test tube, purged with argon, and heated at 90 °C for 24 h. As a result, the product in the form of a thick brown liquid was formed quantitatively and did not require purification.

^1^H NMR (600 MHz, CDCl_3_) δ, ppm: 10.23 (s, 1H), 7.62–7.59 (m, 1H), 7.54–7.51 (m, 1H), 4.37 (t, *J* = 7.0 Hz, 2H), 3.99 (s, 3H), 2.18 (td, J = 6.9, 2.9 Hz, 2H), 2.08-1.99 (m, 3H). ^13^C{^1^H} NMR (151 MHz, CDCl_3_) δ, ppm: 137.60, 123.66, 122.43, 81.50, 70.69, 48.46, 36.53, 28.56, 15.32.

*5,6-dichloro-2-(hex-5-yn-1-yl)phtalimide*. In a test tube, 6-chloro-1-hexyne (0.43 mmol, 0.052 mL), phthalimide (0.5 mmol), K_2_CO_3_ (0.43 mmol, 0.0594 g), and KI (0.0010 g) were added, after which they were dissolved in 0.3 mL of DMF. The reaction was carried out at 70 °C for 16 h. After completing the synthesis, the solution was cooled to room temperature, and 0.5 mL of water was added. Then, the reaction product was extracted with diethyl ether (4 × 10 mL). The collected organic fraction was dried over MgSO_4_, filtered, and concentrated on a rotary evaporator. The resulting mixture was subjected to flash vacuum chromatography with a mixture of petroleum ether and dichloromethane. The isolated yield of the product in the form of a white powder was 63%.

^1^H NMR (600 MHz, CDCl_3_) δ, ppm: 7.91 (s, 2H), 3.71 (t, *J* = 7.2 Hz, 2H), 2.25 (td, *J* = 7.0, 2.6 Hz, 2H), 1.95 (t, *J* = 2.6 Hz, 1H), 1.85–1.77 (m, 2H), 1.60–1.53 (m, 2H). ^13^C{^1^H} NMR (151 MHz, CDCl_3_) δ, ppm: 166.46, 138.99, 131.37, 125.45, 83.69, 69.01, 38.06, 27.60, 25.73, 18.07.

### 4.3. Model Reaction of Thiol **1a** with Alkyne **2a** in Petroleum Medium

Catalyst (IMes)Pd(acac)Cl (0.0055 g, 10^−2^ mmol) was added to the test tube and washed with 1 mL (0.8746 g) of petroleum. Then, pentanethiol-1 **1a** (1 mmol, 0.102 mL) and alkyne **2a** (1 mmol, 0.134 g) were added, and the test tube was closed. The reaction was stirred at 100 °C for 4 h.

A similar reaction procedure was followed for 0.1 M, 0.05 M, and 0.01 M substrate concentrations.

The ESI-HRMS spectrum was registered from a 5 µL aliquot diluted in CH_3_CN 1000 times for the 1 M solution, 100 times for the 0.1 M solution, 50 times for the 0.05 M solution, and 10 times for the 0.01 M solution.

The vinyl sulfide **3a** was isolated by column chromatography with EtOH/EtOAc gradient elution.

^1^H NMR (300 MHz, CDCl_3_) δ, ppm: 7.48 (s, 1H), 7.06 (br. s, 1H), 6.91 (br. s, 1H), 5.02 (s, 1H), 4.75 (s, 1H), 3.94 (t, *J* = 7.0 Hz, 2H), 2.69 (t, *J* = 7.3 Hz, 2H), 2.23 (t, *J* = 7.3 Hz, 2H), 2.09–1.98 (m, 2H), 1.70–1.59 (m, 2H), 1.41–1.28 (m, 4H), 0.90 (t, *J* = 7.0 Hz, 3H).

^13^C NMR (151 MHz, CDCl_3_) δ, ppm: 144.24, 137.33, 129.56, 118.90, 106.74, 45.94, 34.42, 31.40, 31.37, 29.85, 28.02, 22.40, 14.08.

### 4.4. Hydrothiolation of Alkyne **2a** in Petroleum Medium

A 25 mL test tube with a stirring bar was loaded with 0.0016 g (3 × 10^−3^ mmol) (IMes)Pd(acac)Cl followed by 5 mL (4.373 g) petroleum. Then, alkyne **2a** (0.067 g, 0.5 mmol), thiophenol (0.010 mL, 0.1 mmol), benzylthiol (0.012 mL, 0.1 mmol), cyclohexylthiol (0.012 mL, 0.1 mmol), 1-pentanethiol (0.012 mL, 0.1 mmol), and *tert*-butyl thiol (0.011 mL, 0.1 mmol) were added. The test tube was closed, and the reaction was stirred overnight at 100 °C.

In the reaction with natural thiols, alkyne **2a** (0.013 g, 0.1 mmol) was added to a solution of (IMes)Pd(acac)Cl (0.0005 g, 10^−3^ mmol) in 1 mL of petroleum and stirred overnight at 100 °C.

The ESI-HRMS spectrum was registered from a 5 µL aliquot diluted with 1 mL of CH_3_CN.

## Figures and Tables

**Table 1:** Dependence of signal intensity in the ESI-MS spectrum on the concentration of substrates in petroleum.

Thiol 1a Concentration, M	Thiol 1a Concentration, %	Observed Intensity of Alkyne 2a Signal in ESI-HRMS	Observed Intensity of Product 3a Signal in ESI-HRMS
1	8	1.25 × 10^6^	1.25 × 10^6^
0.1	1	1.25 × 10^6^	1.25 × 10^6^
0.05	0.2	1.2 × 10^6^	1.25 × 10^6^
0.01	0.1	1.2 × 10^6^	6 × 10^5^

## Data Availability

Data are contained in the article and Supplementary Materials.

## Supplementary Materials

The following are available online. Supplementary materials contain: the experimental procedure of the model reaction of 1a with labeled alkynes, product 3 NMR yields (Table S1), and results of the ESI-HRMS study (Figure S1); Figure S2—1H NMR spectrum of the model reaction of 2a with 1 M solution of thiol 1a in petroleum; Figure S3—ESI-MS spectra of the model reaction mixtures in petroleum with different substrates concentrations; Figure S4—Experimentally detected and theoretical ESI-(+)MS spectrum of the reaction mixture of five thiols and alkyne 2a in petroleum after 18 h at 100 °C; Figures S5–S9—Enlarged selected area of Figure S4; Figure S10—Enlarged selected area of Figure 3A; Figure S11—Experimentally detected and theoretical ESI-(+)MS spectrum of the reaction mixture of naturally occurring thiols in petroleum with alkyne 2a after 18 h at 100 °C.
