# Supplementary material for: ESI-MS Analysis of Thiol-yne Click Reaction in Petroleum Medium
Source: Molecules. 2021 May 13;26(10):2896. doi: 10.3390/molecules26102896 (PMC8153120; doi:10.3390/molecules26102896)
Supplement: Supplementary file 1 [file molecules-26-02896-s001.zip › molecules-1190280-supplementary.pdf]

molecules

MDPI

Supplementary Materials

ESI-MS Analysis of Thiol-yne Click Reaction in Petroleum Medium

Evgeniya S. Degtyareva, Julia V. Burikyna and Valentine P. Ananikov *

Zelinsky Institute of Organic Chemistry, Russian Academy of Sciences, Leninsky Prospect 47, Moscow, 119991, Russia; http://AnanikovLab.ru; ed@ioc.ac.ru (E.S.D.); ivanova@ioc.ac.ru (J.V.B.); val@ioc.ac.ru (V.P.A.)
* Correspondence: val@ioc.ac.ru

Contents

1. The model reaction of ESI-labeled alkynes with thiol 1a........1

1.1. The model reaction of pentane thiol 1a addition to alkynes in petroleum ether........1

1.2. Development of the method for the analysis of the alkyne hydrothiolation reaction in low concentrations..3

References........9

1. The model reaction of ESI-labeled alkynes with thiol 1a

1.1. The model reaction of pentane thiol 1a addition to alkynes in petroleum ether

The test tube was filled with 0.0006 g (0.001 mmol) (IMes)Pd(acac)Cl and 1 mL of petroleum ether followed by 0.012 mL (0.1 mmol) of pentanethiol-1 and 0.1 mmol of the corresponding alkyne. After closing the tube, the reaction mixture was stirred at 100 ° C for 1 h. The ^{1}H NMR spectra were registered directly from 0.050 mL of reaction mixture diluted with CD_2Cl_2 or $CDCl_3$. The ESI-HRMS spectra, obtained from the reaction mixtures and diluted 200 times, are demonstrated in Figure S1.

Table S1. Influence of the alkyne choice on product **3** yields.

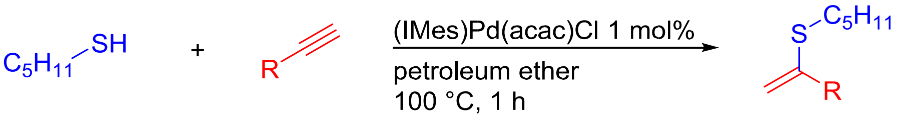

1a **2** **3**

ESI-labeled alkyne 2	Alkyne conversion to 3, % [1]	ESI-labeled alkyne 2	Alkyne conversion to 3, % [1]
1-(pentyn-4-yn-1-yl)1*H*-imidazole (**2a**)	18	N-(5-Hexynyl)phthalimide	30
3-methyl-1-(pent-4-yn-1-yl)-1H-imidazol-3-ium chloride	3	(5,6-dichlori-2-hex-5yn-1-yl)phtalimide	13
but-3-yn-1-yltriphenylphosphonium bromide	0		

[1] The alkyne conversion was determined by ^{1}H NMR.

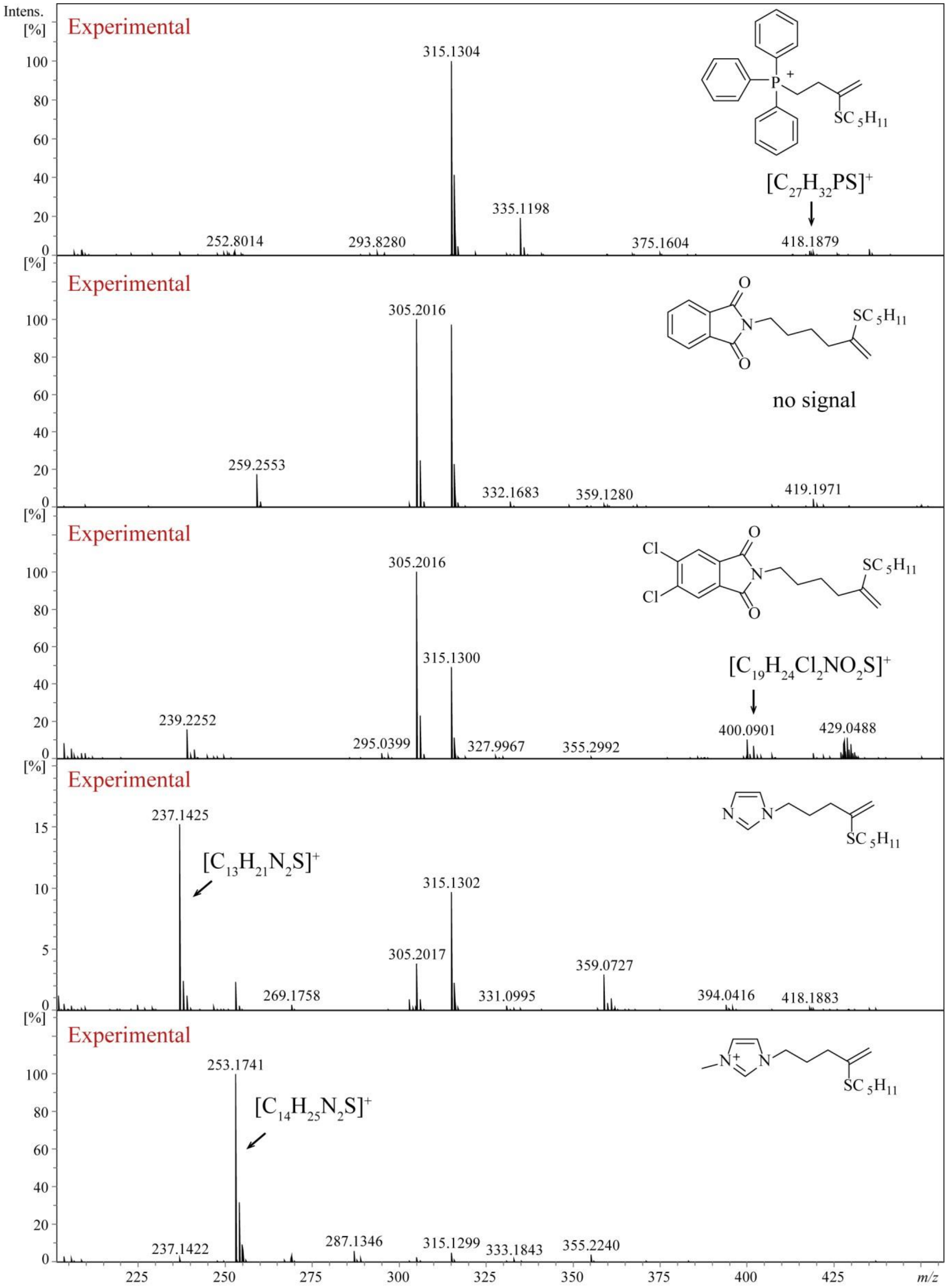
Intens. [%]
Experimental
315.1304
335.1198
252.8014
293.8280
375.1604
$[C_{27}H_{32}PS]^+$
418.1879
SC_5H_{11}
Experimental
305.2016
259.2553
332.1683
359.1280
419.1971
no signal
SC_5H_{11}
Experimental
305.2016
315.1300
239.2252
295.0399
327.9967
355.2992
$[C_{19}H_{24}Cl_2NO_2S]^+$
400.0901
429.0488
Cl
Cl
SC_5H_{11}
Experimental
237.1425
$[C_{13}H_{21}N_2S]^+$
315.1302
305.2017
359.0727
269.1758
331.0995
394.0416
418.1883
SC_5H_{11}
Experimental
253.1741
$[C_{14}H_{25}N_2S]^+$
237.1422
287.1346
315.1299
333.1843
355.2240
SC_5H_{11}
m/z

.

Figure S1. Results of the ESI-HRMS study of the model reaction with various alkynes in petroleum ether.

1.2. Development of the method for the analysis of the alkyne hydrothiolation reaction in low concentrations

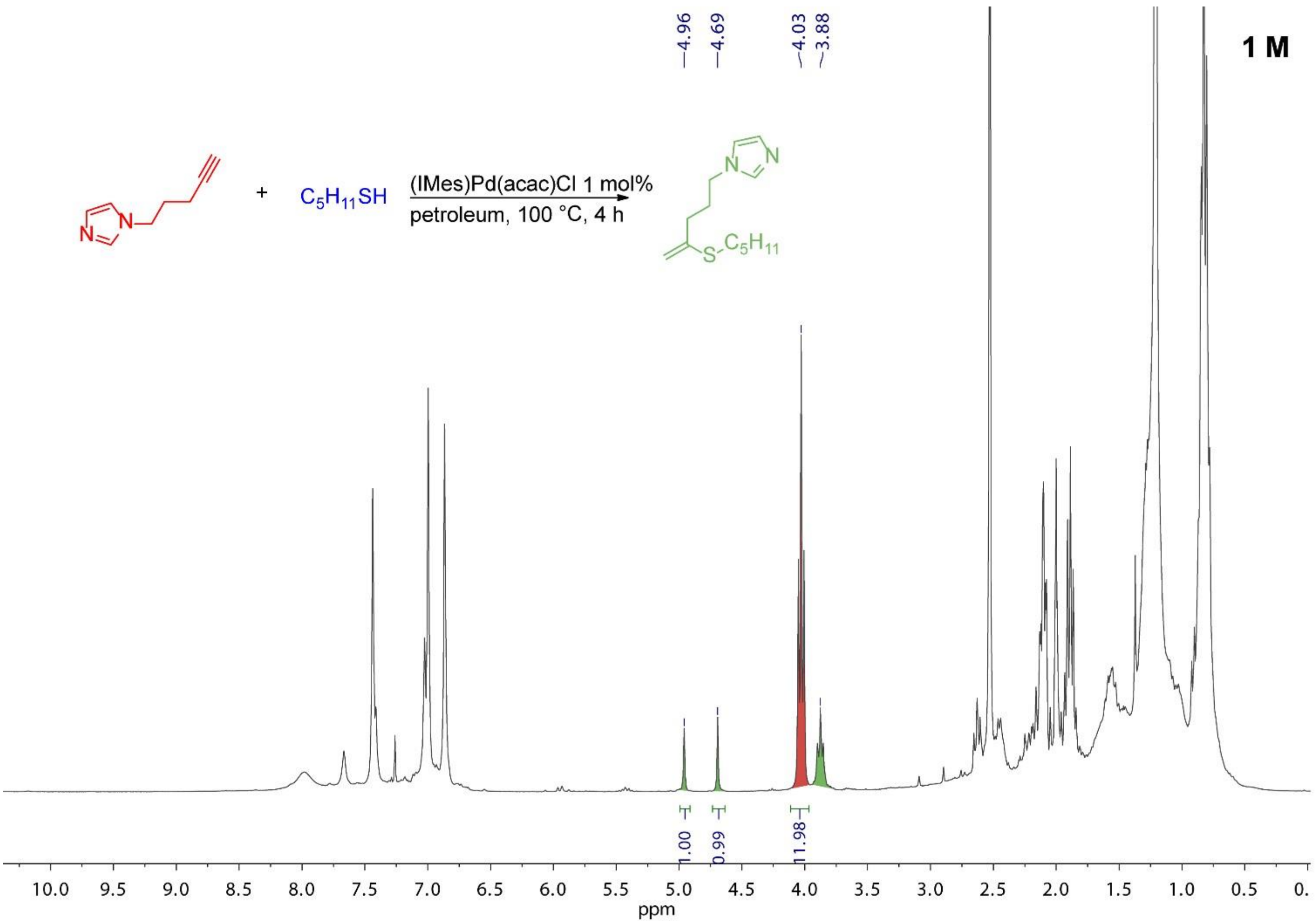

Figure S2. ^{1}H NMR spectrum of the model reaction of **2a** with a 1 M solution of thiol **1a** in petroleum.

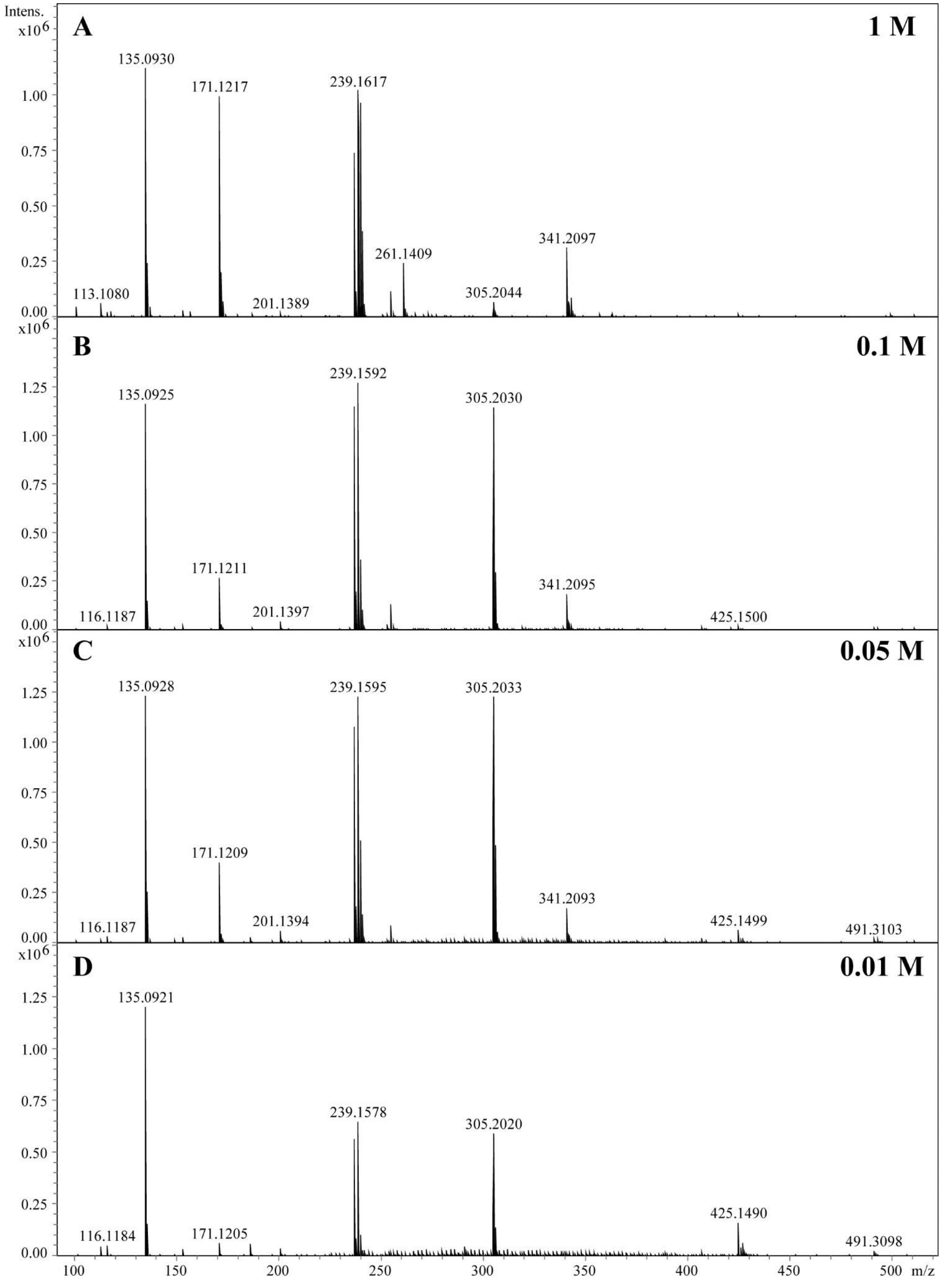

Figure S3. ESI-MS spectra of the model reaction mixtures in petroleum with different substrate concentrations.

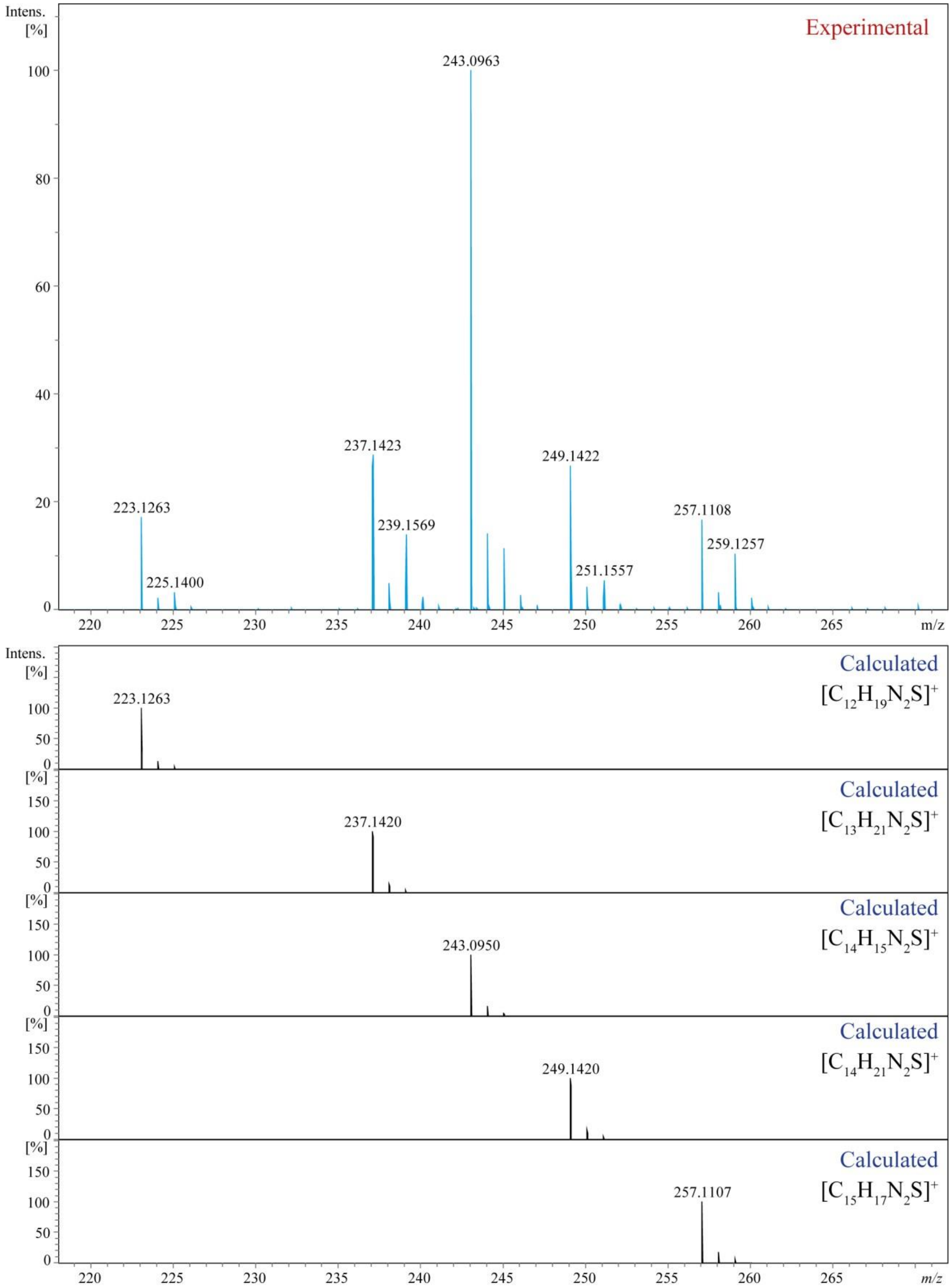

Intens. [%]
Experimental
243.0963
237.1423
249.1422
223.1263
239.1569
257.1108
259.1257
251.1557
225.1400
m/z
Calculated
[C12H19N2S]+
223.1263
Calculated
[C13H21N2S]+
237.1420
Calculated
[C14H15N2S]+
243.0950
Calculated
[C14H21N2S]+
249.1420
Calculated
[C15H17N2S]+
257.1107
m/z

Figure S4. Experimentally detected and theoretical ESI-(+)MS spectrum of the reaction mixture of five thiols and alkyne **2a** in petroleum after 12 h at 100 °C.

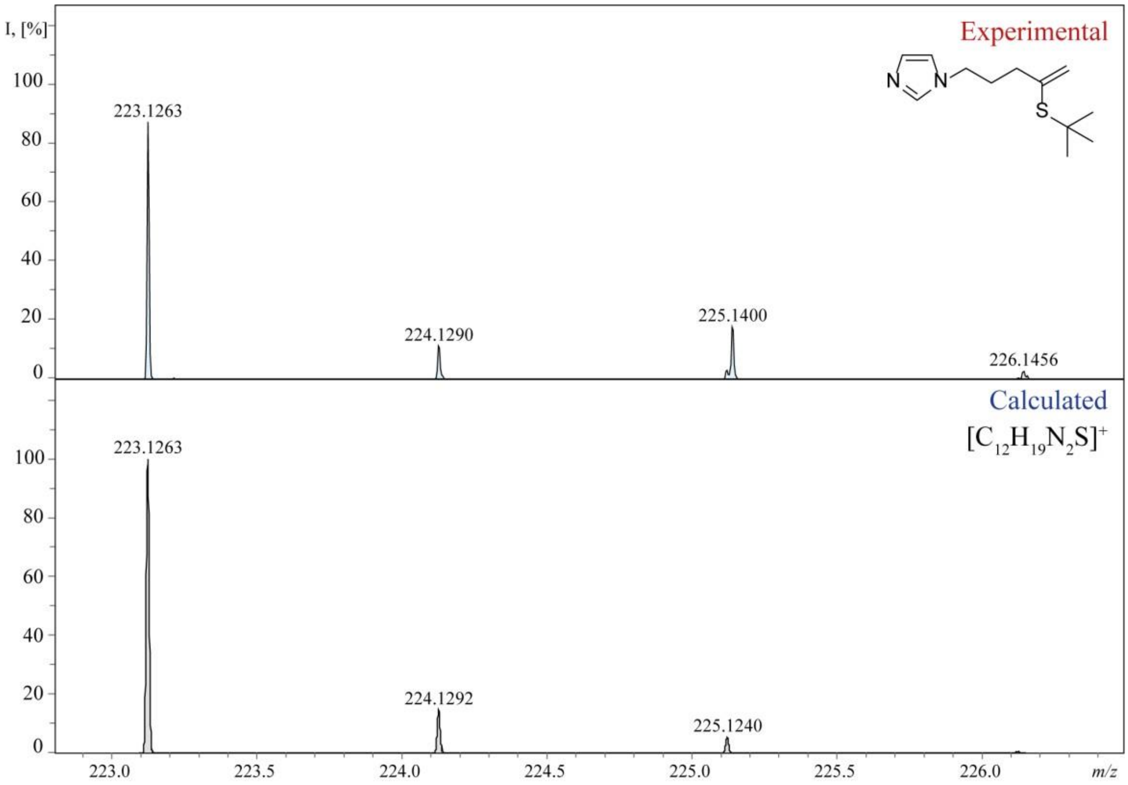

Figure S5. Extended Figure S4. Experimentally detected and theoretical ESI-(+)MS spectrum of 1-(4-(tert-butylthio)pent-4-en-1-yl)-1H-imidazole; main experimental peak $[MH]^+$= 223.1271 Da, calculated for $C_{12}H_{19}N_2S$ = 223.1263 Da, Δ = 0.0 ppm.

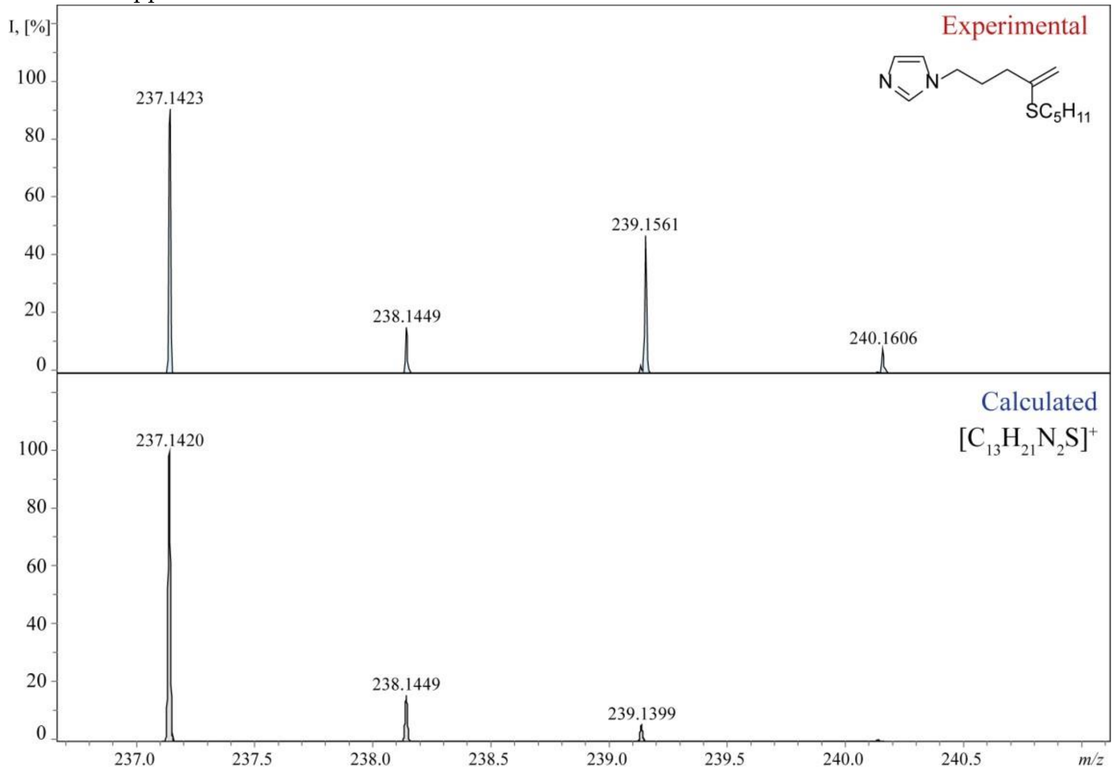

Figure S6. Extended Figure S4. Experimentally detected and theoretical ESI-(+)MS spectrum of **3a**; main experimental peak $[M-H]^+$= 237.1431 Da, calculated for $C_{13}H_{21}N_2S$ = 237.1420 Da, Δ = 1.2 ppm.

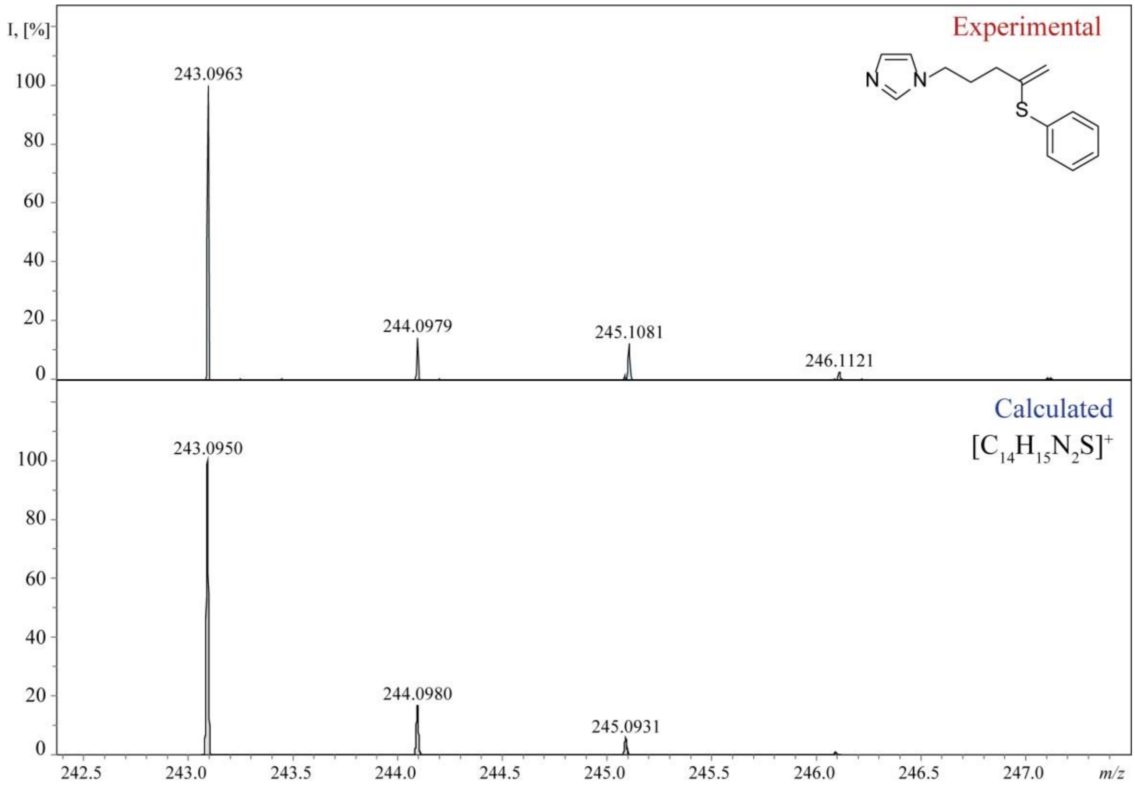

Figure S7. Extended Figure S4. Experimentally detected and theoretical ESI-(+)MS spectrum of 1-(4-(phenylthio)pent-4-en-1-yl)-1H-imidazole; main experimental peak $[M-H]^+$= 243.0963 Da, calculated for $C_{14}H_{15}N_2S$ = 243.0950 Da, Δ = 5.3 ppm.

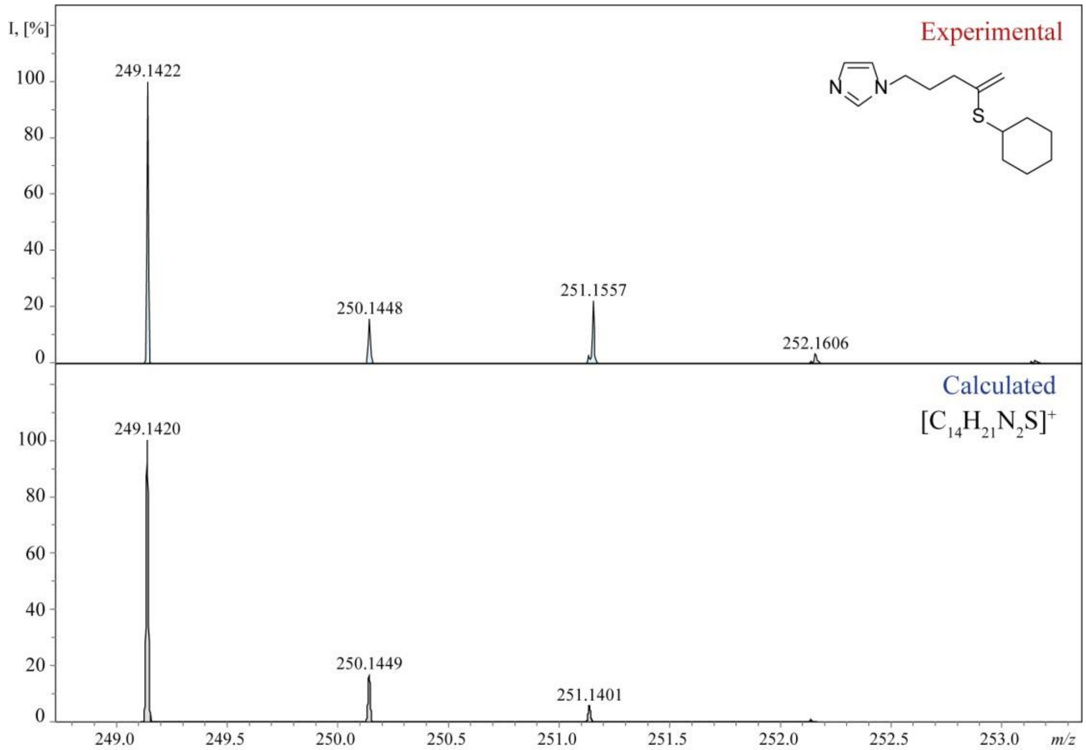

Figure S8. Extended Figure S4. Experimentally detected and theoretical ESI-(+)MS spectrum of 1-(4-(cyclohexylthio)pent-4-en-1-yl)-1H-imidazole; main experimental peak $[MH]^+$= 249.1430 Da, calculated for $C_{14}H_{21}N_2S$ = 249.1420 Da, Δ = 0.1 ppm.

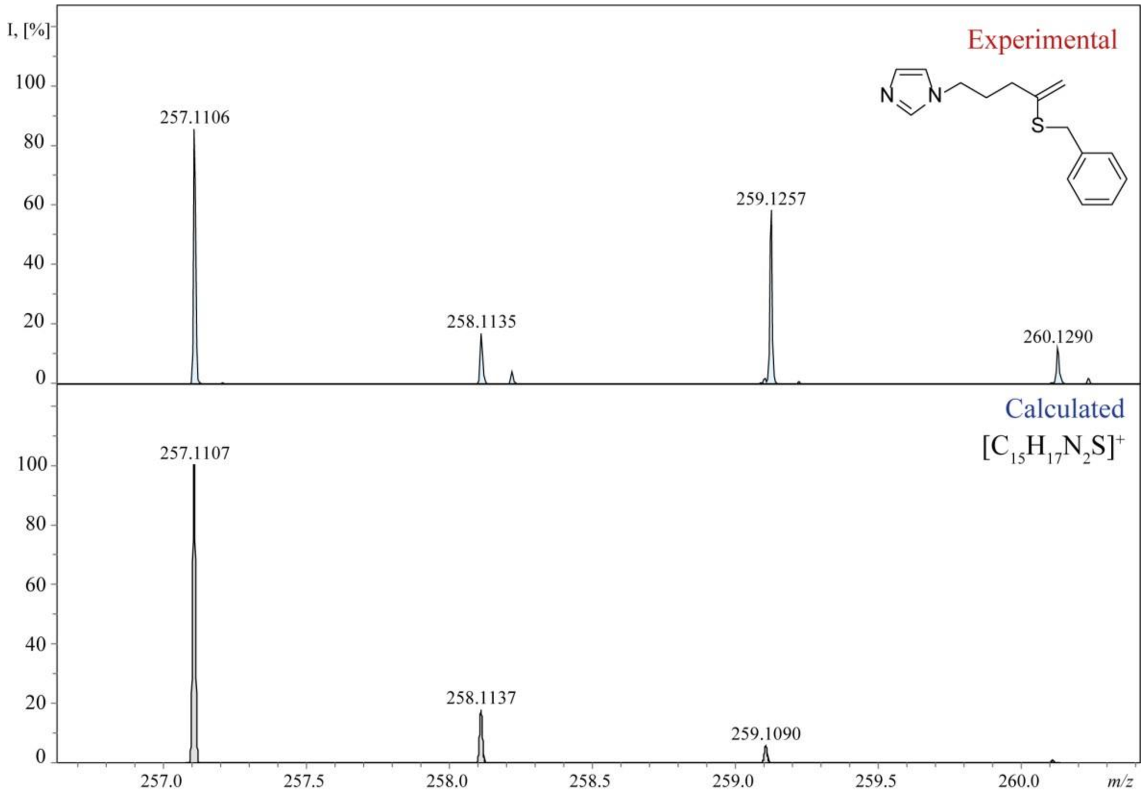

Figure S9. Extended Figure S4. Experimentally detected and theoretical ESI-(+)MS spectrum of 1-(4-(benzylthio)pent-4-en-1-yl)-1H-imidazole; main experimental peak $[M-H]^{+}$= 257.1116 Da, calculated for $C_{15}H_{17}N_2S$ = 257.1107 Da, Δ = 0.4 ppm.

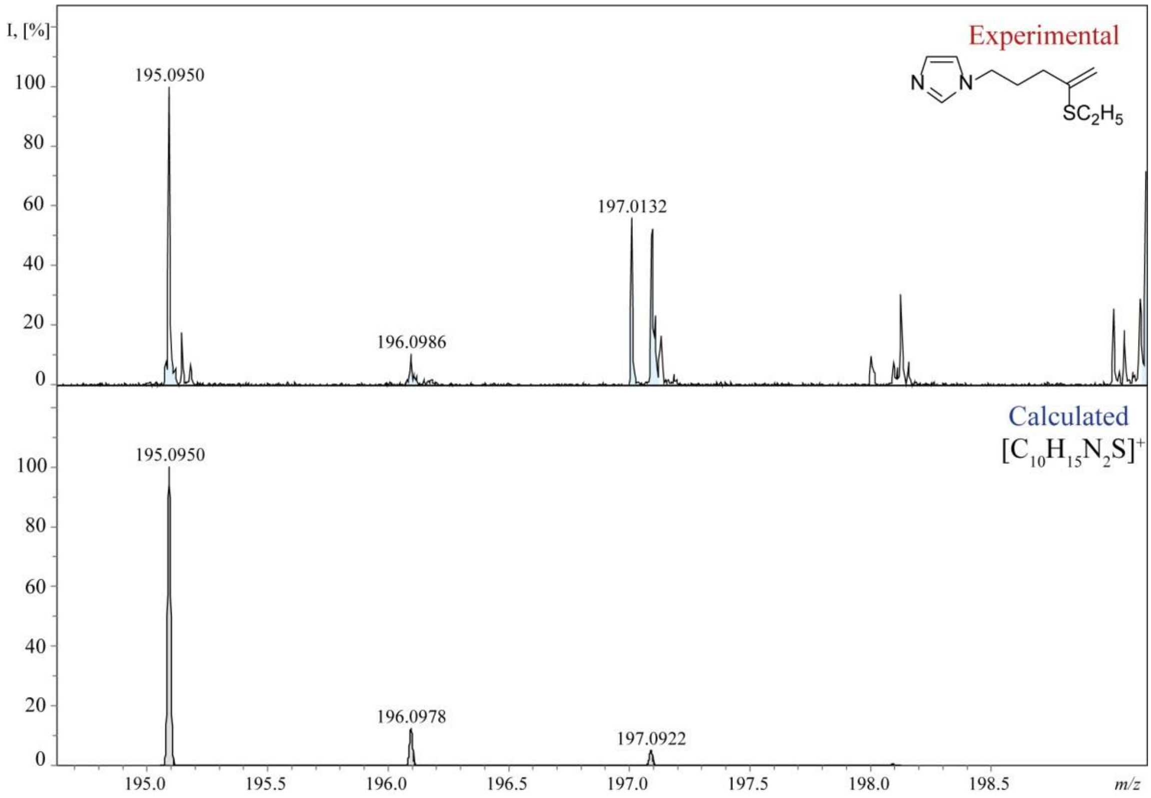

Figure S10. Extended Figure S4. Experimentally detected and theoretical ESI-(+)MS spectrum of 1-(4-(ethylthio)pent-4-en-1-yl)-1H-imidazole; main experimental peak $[M-H]^{+}$= 195.0950 Da, calculated for $C_{10}H_{15}N_2S$ = 195.0950 Da, Δ = 0.0 ppm.

References

1. Perl, N.R.; Ide, N.D.; Prajapati, S.; Perfect, H.H.; Duron, S.G.; Gin, D.Y. Annulation of Thioimidates and Vinyl Carbodiimides to Prepare 2-Aminopyrimidines, Competent Nucleophiles for Intramolecular Alkyne Hydroamination. Synthesis of (−)-Crambidine. *J. Am. Chem. Soc.* **2010**, *132*, 1802–1803, doi:10.1021/ja910831k.
2. Marion, N.; De Frémont, P.; Puijk, I.M.; Ecarnot, E.C.; Amoroso, D.; Bell, A.; Nolan, S.P. N-Heterocyclic Carbene–Palladium Complexes [(NHC)Pd(acac)Cl]: Improved Synthesis and Catalytic Activity in Large-Scale Cross-Coupling Reactions. *Adv. Synth. Catal.* **2007**, *349*, 2380–2384, doi:10.1002/adsc.200700195.
3. Yu, T.-B.; Bai, J.Z.; Guan, Z. Cycloaddition-Promoted Self-Assembly of a Polymer into Well-Defined β Sheets and Hierarchical Nanofibrils. *Angew. Chem. Int. Ed.* **2009**, *48*, 1097–1101, doi:10.1002/anie.200805009.
